# Elevated Carbon Dioxide Levels Decreases Cucumber Mosaic Virus Accumulation in Correlation with Greater Accumulation of rgs-CaM, an Inhibitor of a Viral Suppressor of RNAi

**DOI:** 10.3390/plants10010059

**Published:** 2020-12-29

**Authors:** Huijuan Guo, Panpan Ge, Jiahui Tong, Yanjing Zhang, Xinhong Peng, Zihua Zhao, Feng Ge, Yucheng Sun

**Affiliations:** 1 State Key Laboratory of Integrated Management of Pest Insects and Rodents, Institute of Zoology, Chinese Academy of Sciences, Beijing 100101, China; guohj@ioz.ac.cn (H.G.); gepanpan@ioz.ac.cn (P.G.); tongjh@cau.edu.cn (J.T.); 17863800842@163.com (Y.Z.); m15270803147@163.com (X.P.); 2 CAS Center for Excellence in Biotic Interactions, Chinese Academy of Sciences, Beijing 100049, China; 3 Department of Entomology, College of Plant Protection, China Agricultural University, Beijing 100193, China; zhzhao@cau.edu.cn; 4 Institute of Plant Protection, Chinese Academy of Agricultural Science, Beijing 100193, China

**Keywords:** cucumber mosaic virus, elevated CO_2_, RNA silencing, salicylic acid signaling pathway, 2b protein

## Abstract

Plant viruses cause a range of plant diseases symptoms that are often responsible for significant crop production losses and the severity and spread of the symptoms may be affected by climate change. While the increase in anthropogenic activities has caused a critical problem of increased CO_2_ levels in the atmosphere, these elevated CO_2_ levels have been reported to reduce virus disease severity in some plant species. In such instances, it is not clear if the plant defense mechanisms are being enhanced or virus-mediated mechanisms to overcome plant resistance are being defeated. Additionally, a few studies have been attempted in this area to determine if reduced disease is the norm or the exception under enhanced CO_2_ levels. In the present study, the effects of elevated CO_2_ levels (750 ppm vs. 390 ppm) on RNAi-mediated resistance of *Nicotiana tabacum* against the cucumber mosaic virus (CMV), and the activity of viral suppressor of RNAi (VSR) 2b protein of CMV were evaluated. On the one hand, our results showed that elevated CO_2_ decreased the transcription of dicer-like protein 2 (*DCL2*), *DCL4*, and argonaut 1 (*AGO1*) genes with functions related to RNAi-mediated resistance when infected by CMV, which is contradictory with the decreased CMV copy numbers under elevated CO_2_. On the other hand, we found that elevated CO_2_ increased the calcium concentration and expression of the calcium-binding protein rgs-CaM in tobacco plants when infected by CMV, which directly weakened the function of 2b protein, the VSR of CMV, and therefore decreased the infection efficiency of the virus and suppressed the severity of CMV in tobacco plants under elevated CO_2_. This study provides molecular insights into the ecological implications underlying the development of prevention strategies against plant virus infection in the context of climate change.

## 1. Introduction

The atmospheric CO_2_ concentration has risen from 280 ppm to 400 ppm since the industrial revolution, and is predicted to reach 540–900 ppm by the end of this century [1]. Elevated CO_2_ alters the photosynthesis, carbohydrate assimilation, transpiration rate, and other aspects [2,3], which could substantially change the primary and secondary metabolism of plant tissues; therefore, it affects the interactions with pathogens and herbivorous insects [4]. The response of herbivorous insects to elevated CO_2_ has been well studied [5,6]. In contrast, the response of plant pathogens to elevated CO_2_ have been little studied.

The effects of elevated CO_2_ on the severity of plant diseases caused by pathogens differ among pathogen species [7]. Previous studies showed that elevated CO_2_ increased plant susceptibility to several fungal pathogens [8,9], but increased resistance to certain bacterial pathogens [10,11]. The cross-talk between the salicylic acid (SA) and jasmonic acid (JA) signaling pathways was vital for plant resistance against bacterial and fungal pathogens under elevated CO_2_ [11,12]. Viruses are obligate intracellular parasites and extensively use host cells for their replication and infection, where host plants evolve different resistance strategies against viruses when compared with bacterial and fungal pathogens. Plant defense responses are based on RNA interference (RNAi)-mediated resistance against viral pathogens [13,14]. Previous studies have found that elevated CO_2_ increased plant resistance of artificially inoculated plants against and tomato yellow leaf curl virus (TYLCV) and potato virus Y [15,16]. It is unknown whether the increased resistance can be attributed to an enhancement of the antiviral RNAi levels.

Virus-induced RNAi-mediated resistance includes initiation, amplification, and translational inhibition [17,18]. Silencing is initiated when the triggered dsRNA is firstly recognized by a host dicer-like (DCL) protein, and then cut into 21–24 nucleotide virus short interfering RNAs (vsiRNAs). Cellular RNA-dependent RNA polymerases (RDRs) that use single-stranded RNA (ssRNA) to amplify long, perfect dsRNA [19], which serve as a substrate for the DCL-dependent formation of secondary vsiRNAs [17]. These vsiRNAs are degraded by the core components of the RNA-induced silencing complex (RISC) argonaut (AGO) protein [18]. In contrast, plant viruses often encode viral suppressors of RNAi (VSRs) to suppress the plant RNA-silencing pathway [14]. For example, the 2b protein of cucumber mosaic virus (CMV) suppresses the plant RNA-silencing pathway through interaction with the AGO1 protein PAZ domain [20]. Once 2b protein is mutated, *Nicotiana benthamiana* and *Arabidopsis* do not exhibit the disease symptoms. It seems that the activity of 2b protein in CMV is a determinant for the infection efficiency of the virus. Moreover, some VSR activity, including 2b protein in CMV can be suppressed by rgs-CaM, a calmodulin in *N. benthamiana* [21]. Furthermore, the free-calcium concentration and downstream calmodulin gene expression were increased under elevated CO_2_ [22]. Therefore, it was speculated that the effects of elevated CO_2_ on host calcium concentration and calmodulin gene expression may affect the VSR activity and the interaction between plants and viruses. Thus, the effects of elevated CO_2_ on the process of plant RNAi-mediated resistance and the function of the VSR of viruses should be evaluated.

Widespread evidence shows that elevated CO_2_ decreases the severity of plant viruses in terms of disease incidence, severity, and viron copy numbers [15,16]. It was conceived that the RNA-silencing ability of plants may be enhanced by elevated CO_2_ when attacked by a plant virus. Therefore, we propose two hypotheses underlying the positive effects of elevated CO_2_ on plant RNA-silencing processes against viruses: (1) elevated CO_2_ enhances the key genes involved in plant RNA-silencing processes against viruses and (2) elevated CO_2_ activates the host factor calmodulin to suppress the VSR activity and subsequently relieve the RNA-silencing ability of host plants. We used stably transformed *N. tabacum* plants silenced in rgs-CaM, as well as CMV and its VSR 2b protein mutant CMVΔ2b to experimentally test these hypotheses. Our specific goals were to determine: (1) how elevated CO_2_ affected CMV severity associated with tobacco plants; (2) whether elevated CO_2_ directly affected the RNA-silencing related key gene expression of wild-type plants when infected by CMV; and (3) whether elevated CO_2_ affected the 2b protein expression of CMV by altering the calcium signal and downstream calmodulin expression and whether the changes in 2b protein would, in turn, affect the RNA-silencing ability of plants.

## 2. Materials and Methods

### 2.1. Atmospheric CO_2_ Treatments

The present study was performed in six closed-dynamic CO_2_ chambers (68 cm long, 68 cm wide, and 185 cm high, Safe PRX-450B (Safe, Ningbo, China) according to the previous method [23]. The chambers were maintained at 22 ± 0.8 °C and 70 ± 2% relative humidity (RH), with a 16 h light and 8 h dark photoperiod and with 30,000 LX of active radiation supplied by 18 fluorescent lamps (60 W) during the light period.

Two CO_2_ levels, 400 ± 20 ppm (current ambient level) and 700 ± 20 ppm (predicted level at the end of this century) were applied. Three chambers were used for each CO_2_ treatment. Elevated CO_2_ concentrations were monitored and adjusted with an infrared CO_2_ transmitter (Ventostat 8102, Telaire Company, Goleta, CA, USA) once every 2 s to maintain stable CO_2_ concentrations.

### 2.2. Host Plants and Viruses

We generated stably transgenic irrgs-CaM tobacco plants through *Agrobacterium*-mediated transformation of *Nicotiana tabacum* (cv. W38) as background plants. In brief, the rgs-CaM (Accession number: AF329729) fragment amplified with forward primer: 5′-ATGGAAAAGTGTCACCGGCT-3′ and reverse primer: 5′-TGTAGCCACTCCCCTCCA TT-3′, was inserted into the pCAMBIA2301 transformation vector in an inverted-repeat orientation ([24]; Figure S1). Hypocotyls from the seedling stage were dipped into the *Agrobacterium* suspension transformation according to [25]. Several independently transformed lines of irrgs-CaM plants that harbored single transgene insertions were identified by the transcript levels of rgs-CaM in these lines were analyzed by RT-qPCR. Two lines, line067 and line098, were selected for further studies based on their efficient knockdown of *rgs-CaM* genes (T0). When seeds were collected, they were screened with hygromycin and used for further experimentation. The T1 seedlings were individually transplanted into plastic pots (12 cm diameter and 14 cm high) according to previous study [26].

The plant virus CMV strain SD-CMV and its 2b gene deletion mutant, Δ2b CMV, was kindly provided by Professor Huishan Guo from the Institute of Microbiology, Chinese Academy of Sciences [27]. After 4 weeks of growth, the plants were randomly divided into two groups for CMV inoculation and determination of the plant response.

### 2.3. CMV Accumulation as Affected by CO_2_ Level in Wild-Type Plants (Group 1)

Thirty 4-week-old wild-type *N. tabacum* per CO_2_ level were inoculated with 5, 50, and 500 μL of purified virions of CMV at a concentration of 200 μg/mL (there are 3–4 plants of per chamber each inoculated virion concentration, and 60 plants in total). After 14 days, the virus accumulation of infected leaves was determined with qPCR. Furthermore, another 30 4-week-old wild-type *N. tabacum* per CO_2_ level were inoculated with 50 μL of purified virions of CMV at a concentration of 200 μg/mL, and the CMV accumulation of systemically-infected leaves were determined with RT-qPCR post-infection for 7, 14, and 21 days (there are 3–4 plants of per chamber each time point, and 60 plants in total), as described below.

### 2.4. Suppression of RNA Silencing by 2b Protein under Elevated CO_2_ (Group 2)

Transgenic *N. benthamiana* plants expressing GFP protein (16c) were provided by Dr. David C. Baulcombe (Sainsbury Laboratory, Norwich Research Park, Norwich, UK) and were previously described [28,29]. For suppression of RNA-silencing assays, 4-week GFP expressed (line 16c) tobacco plants were co-infiltrated with mixed *Agrobacterium* cultures harboring the positive sense GFP (sGFP) expression plasmid with different combinations of 10 μg purified virions of CMV (10 plants) or 10 μg purified virions of CMVΔ2b under both ambient CO_2_ and elevated CO_2_ according to [30]. Four days after injection, the GFP fluorescence was visually detected using a long-wave UV lamp (model B 100 AP, Black Ray, CA, USA). Plants were photographed with a 950 digital camera (Nikon, Tokyo, Japan) mounted with a yellow filters (560 nm). The images were processed electronically using Adobe Photoshop (Adobe, San Jose, CA, USA). Then, the inoculated leaves were collected for determination of the key gene expression of GFP.

### 2.5. The Gene Expression Involved in Plant RNA-Silencing Signaling Pathway

For determination of the key gene expression in RNA-silencing signaling pathway when affected by CO_2_ levels, six CMV infected and six uninfected plants of wild-type (2 plants of each treatment in each of the 3 chambers) under ambient CO_2_ and the same plants under elevated CO_2_ were selected. The RNA Mini Kit (Qiagen, Valencia, CA, USA) was used to isolate total RNA from *N. tabacum* leaves (50 mg from samples stored at −70 °C), and 2 μg of RNA was used to synthesize cDNA. The mRNA amounts of the target genes were quantified using real-time quantitative PCR. The target genes were *DCL2*, *DCL3*, *DCL4*, *RDR1*, *RDR6*, *AGO1*, and *AGO4* (Table S1). Specific primers for the target genes were designed from *N. tabacum* sequences (https://www.ncbi.nlm.nih.gov/nuccore) using Primer Express^®^ software ver. 5.0 (Applied Biosystems, Foster City, CA, USA). Furthermore, the mRNA amounts of 2b gene of single-stranded, positive-sense and tripartite RNA virus CMV in wild-type plant and irrgs-CaM plants were quantified using real-time quantitative PCR according to [26]. The data were represented by six biological replicates, and each biological replicate contained four technical repeats.

### 2.6. CMV Accumulation Determination

The Qiagen RNeasy Plant Mini Kit was used to isolate total RNA from *N. tabacum* leaves (50 mg). In this study, a TaqMan real-time quantitative RT-PCR assay was used for the detection and quantification of the CMV sequences [31]. The primers of 3a gene (F: 5′-TCGCAGCTGGGAAGACTCTA-3′; 5′CGGGAG GGCTCTCAACATTT-3′) generated a 189 bp DNA fragment after amplification. The TaqMan probe (5′-CTCCCGCCGCAATCGGGAGTT-3′) was labeled with fluorescent dyes, 6-carboxyfluoroscein (FAM) on the 5′-end and N, N, N′, N′-tetramethyl-6-carboxyrhodamine (TAMRA) on the 3′-end. The actin gene of *N. tabacum* was used as the internal control. Then, the linear equation of log (CMV virus copy numbers) versus the Ct curve was generated according to [26]. The recombinant plasmid pBI221 was constructed by the PCR product (3a of CMV) insertion into pGEM-T Easy vector (Promega, Fitchburg, WI, USA). The standard curve of the CMV 3a protein gene was obtained by using serial 10-fold diluted plasmids (9.16 × 10^7^ to 9.16 × 10^3^ copies) as templates. For calculation of Y value used for the determination of viral copy number in the tested DNA samples the following equation was applied by using standard formula for the regression analysis calculation: Y = −3.214X + 36.42, it was possible to quantify the viral copy number in the examined samples. Amplifications were performed in 20 μL volumes containing 10 μL of TB Green™ Premix Ex Taq™ 2 (TaKaRa, Shiga, Japan), 2 μL of template, 1 μL of each 10 μM gene-specific primers using the Mx 3500P detection system (Stratagene Corp., La Jolla, CA, USA) as follows: 95 °C for 10 s, followed by 40 cycles of 10 s at 95 °C, and 30 s at 57 °C according to [26]. All samples were run in duplicate by TaqMan Real-Time PCR assay for accuracy of results. After that, a melting curve was performed to verify the specificity of the PCR product. Data were represented by six biological replicates, and each biological replicate contained four technical repeats.

Ten 4-week-old plants of each genotype (including wild-type and irrgs-CaM) in each CO_2_ level were selected to inoculate 0 μg, 1 μg, 10 μg, and 100 μg CMV particles. After CMV infection for 21 d, the plants were harvested for measurement of biomass, stem diameter, and height.

### 2.7. Ca^2+^ Fluorescence Microscopy

The Ca^2+^ Fluorescence of leaves from healthy plants, mock-inoculated, and CMV-inoculated plants under ambient CO_2_ and elevated CO_2_ were determined by using 35S:GCaMP3 *N. benthamiana* plants according to Wang et al. (2020). Fluorescence was analyzed over time for various regions of interest (ROIs) using the Fiji plug-in Time Series Analyzer v2 (University of California, Los Angeles, CA, USA). ΔF/F was calculated according to the equation ΔF/F = (F′ − F)/F, where F was the average baseline fluorescence calculated from the average of F in healthy plant leaves under ambient CO_2_, F′ was the average fluorescence calculated from the treated plant leaves under ambient and elevated CO_2_.

### 2.8. Statistical Analysis

PASW Statistics 18.0 (SPSS Inc., Chicago, IL, USA) was used for statistical analysis. Two-way ANOVAs were used to analyze plant responses and virus copy numbers and 2b protein. In the ANOVA model, CO_2_ level and CMV treatment were the main factors, chambers nested within CO_2_ level and CMV treatment as the random factor. Tukey’s multiple range tests were used to separate means when ANOVAs were significant. All data were checked for normality and equality of residual error variances and appropriately transformed (log or square root) if needed to satisfy the assumptions of the analysis of variance.

## 3. Results

### 3.1. Elevated CO_2_ Decreased the Copy Numbers of CMV

**Figure 1:**
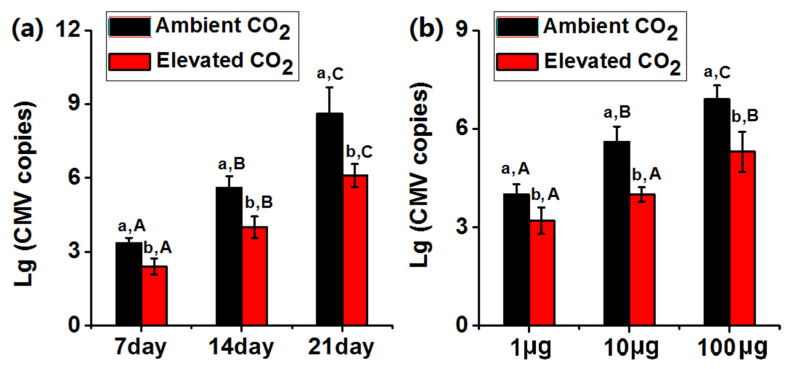
Elevated CO_2_ decreased CMV copy numbers in *Nicotiana tabacum*. (**a**) Accumulation of RNA 3 of CMV in *N. tabacum* post mechanical inoculation with the virus for 7 days, 14 days and 21 days under ambient CO_2_ and elevated CO_2_. (**b**) Accumulation of RNA 3 of CMV in *N. tabacum* post mechanical inoculation with 1, 10 and 100 μg virus for 14 days under ambient CO_2_ and elevated CO_2_. Data represent means ± SE (Tukey’s multiple range test, *p* < 0.05). Different lowercase letters indicate significant differences between ambient CO_2_ and elevated CO_2_ within the same time point or virus concentration. Different uppercase letters indicate significant differences among time points or virus concentrations within the same CO_2_ treatment (*p* < 0.05). Error bars indicate standard errors. Lg: log to base 10.

To investigate whether elevated CO_2_ affected the CMV severity associated with *N. tabacum*, we quantified the CMV copy numbers after different inoculation times and infection times. Elevated CO_2_ decreased CMV copy numbers when inoculated with 10 μg CMV virions post 7 d, 14 d, and 21 d (Figure 1a). Furthermore, elevated CO_2_ significantly decreased CMV copy numbers of wild-type *N. tabacum* when plants artificially inoculated with 1 μg, 10 μg, and 100 μg CMV (Figure 1b).

### 3.2. Elevated CO_2_ Affect Plant Key Genes Expression Associated with Plant Systemic RNA Silencing

**Figure 2:**
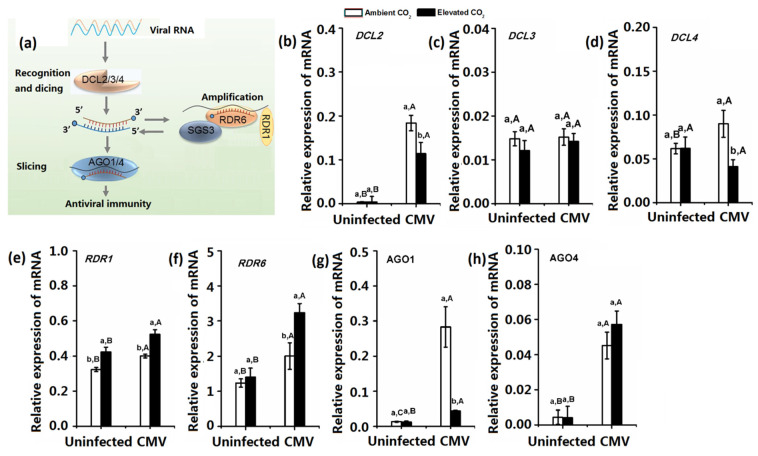
Expression of key genes associated with plant systemic RNA silencing of *N. tabacum* that were grown under ambient CO_2_ and elevated CO_2_ 2 weeks after CMV infection or mock inoculation. (**a**) Representation of the key genes involved in virus-induced RNA silencing occurs in three steps: initiation, amplification and spreading. The expression of (**b**) DCL2, (**c**) DCL3, (**d**) DCL4, (**e**) RDR1, (**f**) RDR6, (**g**) AGO1 and (**h**) AGO4. Data represent means ± SE (Tukey’s multiple range test, *p* < 0.05). Different lowercase letters indicate significant differences between ambient CO_2_ and elevated CO_2_ within the same virus treatment. Different uppercase letters indicate significant differences among virus treatment within the same CO_2_ treatment (*p* < 0.05).

Virus-induced RNA silencing occurs in three steps: initiation, amplification, and translational inhibition [17]. To investigate whether elevated CO_2_ affected the different steps of virus-induced RNA silencing, we determined the key gene expression involved in the initiation (*DCL2*, *DCL3*, *and DCL4*), amplification (*RDR1* and *RDR6*), and translational inhibition (*AGO1* and *AGO4*) steps of RNA silencing under both CO_2_ levels (Figure 2). CMV infection significantly upregulated *DCL2*, *RDR1*, *RDR6*, *AGO1*, and *AGO4* under both CO_2_ levels and DCL4 expression under ambient CO_2_. For the virus uninfected plants, elevated CO_2_ did not affect the gene expression of RNA silencing, except for increasing *RDR1* expression. For the CMV-infected plants, elevated CO_2_ decreased the *DCL2*, *DCL4*, and *AGO1* expression but increased *RDR1* and *RDR6* expression (Figure 2).

### 3.3. Elevated CO_2_ Suppressed the Expression of VSR 2b Protein of CMV

**Figure 3:**
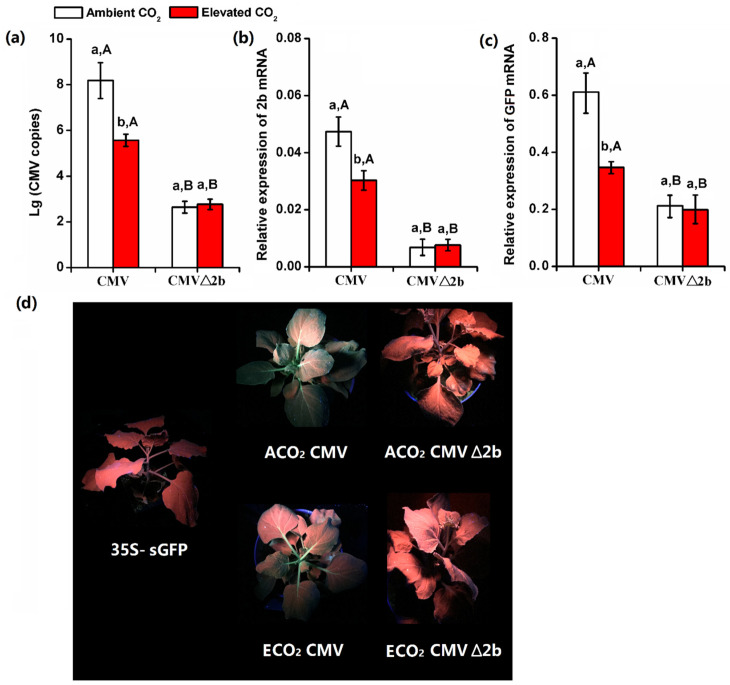
(**a**) CMV viral accumulation and (**b**) the relative expression of 2b mRNA in *N. tabacum* (**c**) the relative expression of GFP in GFP-transgenic *N. benthamiana* 16c plants post agroinoculated with CMV and CMVΔ2b for 14 days under ambient CO_2_ and elevated CO_2_. (**d**) The ability of 2b protein to recover silenced GFP expression in systemic leaves of GFP-transgenic *N. benthamiana* 16c plants. Data represent means ± SE (Tukey’s multiple range test, *p* < 0.05). Different lowercase letters indicate significant differences between ambient CO_2_ (ACO_2_) and elevated CO_2_ (ECO_2_) within the same virus treatment. Different uppercase letters indicate significant differences between virus treatments within the same CO_2_ treatment (*p* < 0.05).

Suppression of long-distance spread of the antiviral RNA silencing represented a viral anti-defensive ability to establish systemic virus infection far beyond the initial invasion sites. For CMV, the VSR 2b protein could suppress the RNA silencing of host plants. Once the 2b protein of CMV was mutated, the CMV copy numbers of wild-type plants were dramatically decreased after inoculated for 14 d (Figure 3a). Elevated CO_2_ significantly decreased CMV copy numbers (Figure 3a,b). We compared the ability of CMV to recover silenced GFP expression in systemic leaves of GFP-transgenic *N. benthamiana* 16c plants, which had been infiltrated with an *Agrobacterium* strain harboring p35S-sGFP (35S: sGFP) to completely silence the GFP expression in the plants under ambient CO_2_ and elevated CO_2_ (Figure 3c,d). The GFP-silenced 16c plants were inoculated with 50 μg/mL CMV or 50 μg/mL CMV△2b. At 10 dpi, elevated CO_2_ decreased the mRNA expression of GFP in CMV infected plants (Figure 3c). The newly emerging leaves of CMV-infected plants under ambient CO_2_ displayed brighter GFP fluorescence, whereas those of CMV-infected plants under elevated CO_2_ showed only weak GFP fluorescence. These results suggested that CMV under ambient CO_2_ was more efficient to suppress systemic RNA silencing in *N. benthamiana* plants as compared with those under elevated CO_2_. Furthermore, once the 2b protein was mutated, CMV△2b -infected plants under both ambient and elevated CO_2_ showed no GFP fluorescence (Figure 3d). This result provided direct evidence that the effects of elevated CO_2_ on the virulence of CMV was correlated with the inability to inhibit RNA silencing in tissue systemically-infected with CMV.

### 3.4. Calcium Concentration and Calmodulin Gene Expression of Plants Was Increased by Elevated CO_2_ When Infected by CMV

**Figure 4:**
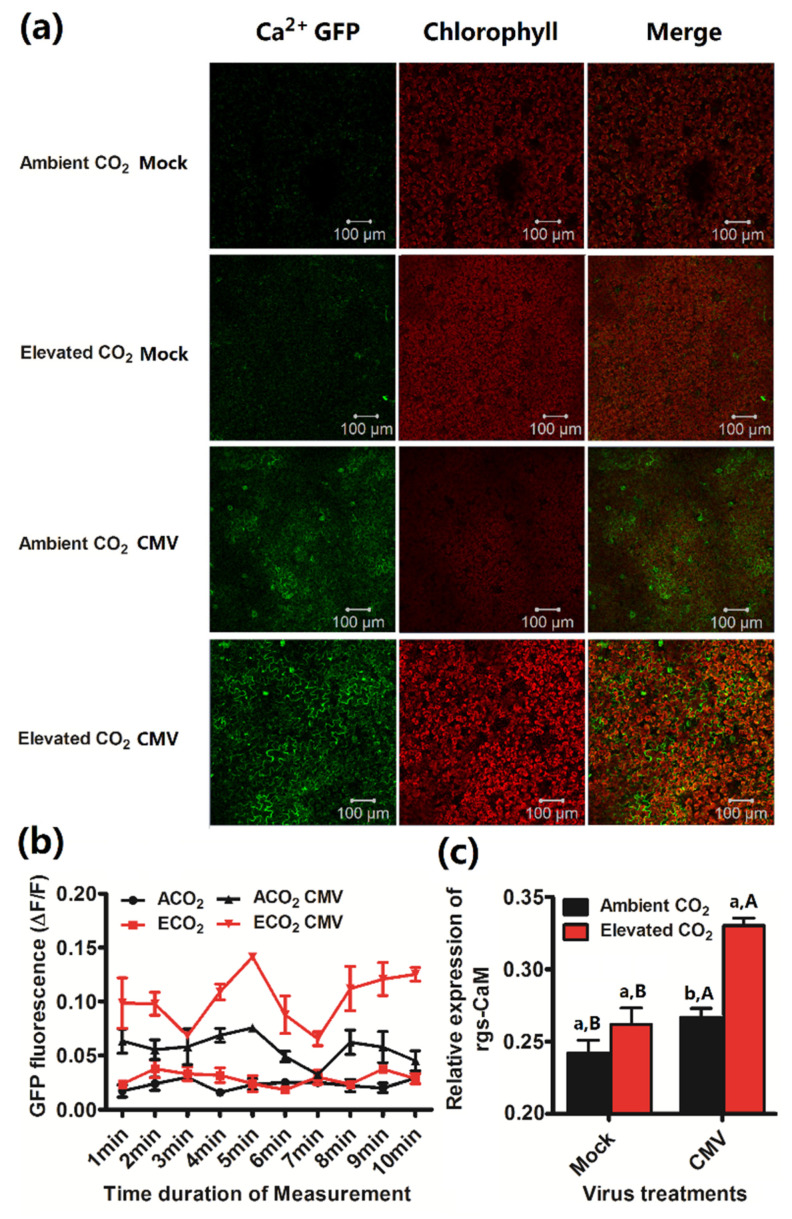
Elevated CO_2_ increased [Ca^2+^]_cyt_ of *N.tabacum* and the relative expression of rgs-CaM when infected by CMV. (**a**) Sub-cellular localization of GFP fluorescence of Ca^2+^ in CMV-infected and mock-inoculated leaves in GCaMP3 transgenic *N. benthamiana* plants post infection for 2 weeks under both CO_2_ levels. Images of the probe (green) and chlorophyll (red) fluorescence emission are shown together with overlay images of these two pictures—100μm. (**b**) Normalized fluorescence (∆F/F) of 35S:GCaMP3 leaves post infection for 2 weeks. ∆F, difference between measured fluorescence of treated plants and baseline fluorescence (F) of healthy plants. (**c**) the relative expression of rgs-CaM mRNA in wild-type *N. tabacum* post agroinoculated with buffer (Mock) or CMV for 14 days under ambient CO_2_ and elevated CO_2_. Data represent means ± SE (Tukey’s multiple range test, *p* < 0.05). Different lowercase letters indicate significant differences between ambient CO_2_ and elevated CO_2_ within the same virus treatment. Different uppercase letters indicate significant differences between virus treatments within the same CO_2_ treatment (*p* < 0.05).

To investigate whether elevated CO_2_ affected the calcium concentration changes in the leaf cytoplasm, we used a transgenic *N. benthamiana* plants expressing the GFP-based Ca^2+^ sensor GCaMP3, to show the [Ca^2+^]cyt changes in epidermal and mesophyll cells of plants (Figure 4a). We found that virus infection induced Ca^2+^ fluorescence under both CO_2_ levels. There was no significant difference in Ca^2+^ fluorescence in the mock-inoculated plants grown under ambient CO_2_ and elevated CO_2_ (Figure 4a,b). Once the plant was infected by CMV, elevated CO_2_ significantly increased Ca^2+^ fluorescence in the virus-inoculated plants (Figure 4b). Furthermore, we also determined the rgs-CaM gene expression and found that elevated CO_2_ increased the rgs-CaM gene expression of virus-infected plants (Figure 4c).

### 3.5. The CMV Copy Numbers and 2b Gene Expression on Irrgs-CaM Plants under Elevated CO_2_

**Figure 5:**
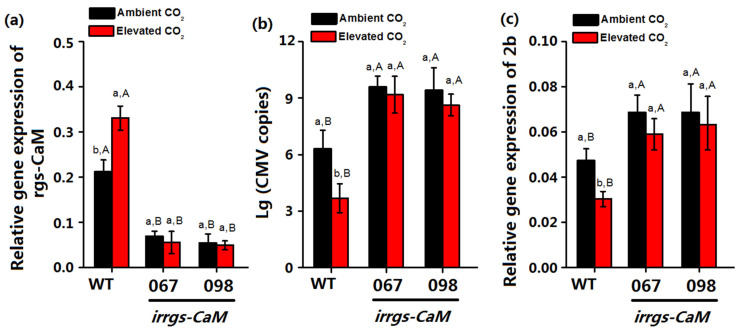
(**a**) relative gene expression of rgs-CaM (**b**) CMV copy numbers and (**c**) the relative expression of 2b mRNA in WT and irrgs-CaM (line067, line098) *N. tabacum* plants post agroinoculated with CMV for 14 days under ambient CO_2_ and elevated CO_2_. Data represent means ± SE (Tukey’s multiple range test, *p* < 0.05). Different lowercase letters indicate significant differences between CO_2_ levels within the same genotype. Different uppercase letters indicate significant differences among three genotype within the same CO_2_ level.

The Ca^2+^ binding protein rgs-CaM could bind CMV 2b protein via its affinity to the negatively charged dsRNA-binding domains of 2b protein and rgs-CaM reinforces antiviral RNA silencing by directing the degradation of 2b protein via autophagy [21]. Thus, the increase of rgs-CaM gene expression under elevated CO_2_ may affect the CMV accumulation. To investigate whether elevated CO_2_ affects the CMV severity via rgs-CaM, we determined the accumulation of RNA 3 of CMV and 2b gene expression in wild-type and irrgs-CaM plants when infected by CMV. The relative mRNA expression of rgs-CaM was significantly lower in irrgs-CaM than wild-type plants regardless of CO_2_ level. For the plants that were artificially inoculated with 10 ng CMV, elevated CO_2_ significantly decreased accumulation of RNA 3 of CMV in CMV-infected wild-type, whereas it did not affect the accumulation of RNA 3 of CMV in CMV-infected irrgs-CaM plants (Figure 5a). Accumulation of RNA 3 of CMV in wild-type plants were lower than those of irrgs-CaM infected plants. Furthermore, elevated CO_2_ significantly decreased 2b gene expression of CMV-infected wild-type and did not affect the 2b gene expression of CMV-infected irrgs-CaM plants. The 2b gene expression of CMV-infected wild-type plants was lower than that of infected irrgs-CaM plants (Figure 5b).

### 3.6. Growth Traits of Plants as Affected by Plant Genotype, CO_2_ Levels, and CMV Infection

**Figure 6:**
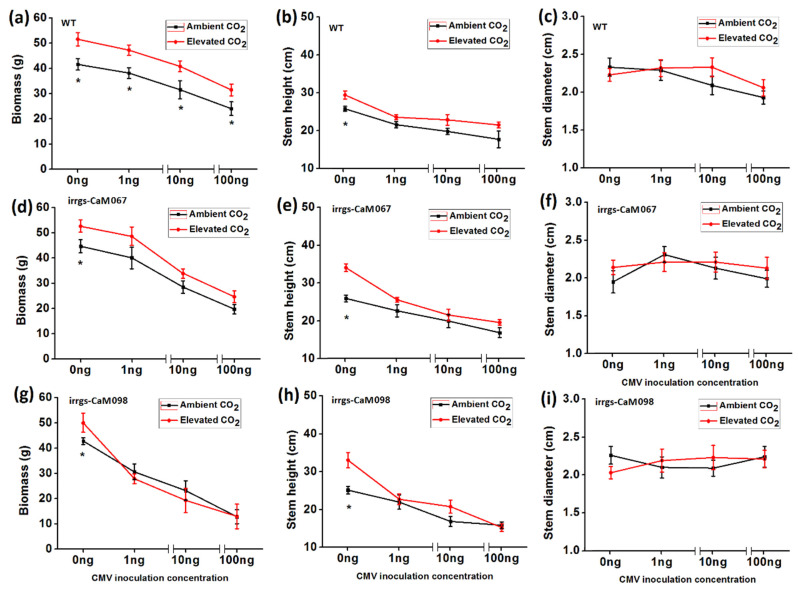
Growth traits of two *N. tabacum* genotypes (WT, irrgs-CaM067, irrgs-CaM098) grown under ambient CO_2_ (ACO_2_) and elevated CO_2_ (ECO_2_) with different CMV inoculation concentration. Data represent means ± SE (Tukey’s multiple range test, *p* < 0.05). (**a**) biomass, (**b**) stem height and (**c**) stem diameter of wild-type (WT) plants. (**d**) biomass, (**e**) stem height and (**f**) stem diameter of irrgs-CaM067 plants. (**g**) biomass, (**h**) stem height and (**i**) stem diameter of irrgs-CaM098 plants.

Elevated CO_2_ increased biomass of wild-type regardless of inoculation with 0, 1, 10 ng, and 100 ng CMV. In contrast, elevated CO_2_ only increased biomass of uninfected irrgs-CaM plants. Furthermore, elevated CO_2_ increased the height of uninfected wild-type and irrgs-CaM plants but did not affect the height of the two genotypes of plants when inoculated with 1, 10 and 100 ng CMV. Elevated CO_2_ did not affect the stem diameter of the two genotypes of plants, regardless of infection by CMV (Figure 6).

## 4. Discussion

In plant and virus interactions, RNA silencing is a general mechanism involved in immunity against viruses; virus deploy VSRs that bind to dsRNA and attenuate RNA silencing, and the arms-race battle has been challenged by the rising atmospheric CO_2_ concentration [32]. The present study showed that elevated CO_2_ levels could increase plant calcium concentration and increase the expression of calcium-binding protein rgs-Cam in CMV-infected tobacco plants. Once a plant is infected by CMV, a previous study showed that the rag-CaM would directly degrade the VSR 2b protein in CMV [21]. We also found that elevated CO_2_ decreased 2b gene expression and subsequently suppress the efficiency of virus infection under elevated CO_2_ conditions.

RNAi-mediated resistance response is a more common defense response to viral invasion. Liu et al. [33] found that the expression of key genes involved in RNAi-mediated resistance, such as *DCL2*, *DCL4*, *AGO1*, *AGO4*, and *RDR1* was upregulated under higher temperatures. Unlike the high temperature, RNAi genes respond in varying ways to CO_2_. We found that the elevated CO_2_ increased RDR1 regardless virus infection and increased RDR6 when infected by CMV. In contrast, elevated CO_2_ did not affect the expression of DCLs, AGOs, and RDR6 in healthy plants and even decreased the expression of *DCL2*, *DCL4*, and *AGO1* in CMV-infected plants. The decrease of key genes in CMV-infected plants may result from the lower virus incidence inducing lower RNAi-mediated resistance under elevated CO_2_. Moreover, the GFP-silencing experiment exhibited that CMV was more efficient in the suppression of systemic RNA silencing in *N. benthamiana* plants and the expression of CMV 2b protein was higher under ambient CO_2_ when compared with those under elevated CO_2_, which indicated that the 2b protein activity of CMV could be directly suppressed under elevated CO_2_. Inconsistent with our study, Del Toro et al., (2015) found that elevated CO_2_ (~970 ppm) did not affected the ability of the 2b protein to relieve the partial silencing of the GFP reporter, compared to ambient CO_2_ at both, protein and transcript levels. Therefore, elevated CO_2_ increased viral titers in leaf disks for CMV. The different result may due to the plant growth condition is different under different high CO_2_ treatment (~700 ppm vs. ~970 ppm) and temperature condition (~22 °C vs. ~25 °C) between the two studies.

The autophagy pathway, one of the cellular protein degradation mechanisms in host plants, is a key regulator in the degradation of the viral VSRs [34]. For example, the autophagy-related gene 8 (*ATG8*) could target the cotton leaf curl Multan virus βC1 protein for degradation. Preventing the interaction between βC1 and ATG8 exacerbated symptoms and enhanced virus accumulation [35]. In the interaction between tobacco and CMV, the VSR 2b protein could also be degraded through the autophagy pathway [21]. This requires an interaction between the 2b and rgs-CaM, a calmodulin-like protein, which is itself destined to autophagic degradation. In the current study, the expression of rgs-CaM in CMV infected plants was upregulated under elevated CO_2_. Once the rgs-CaM was artificially suppressed, the irrgs-CaM plants exhibited higher CMV copy numbers than did the wild-type plants and there was no significant difference in CMV copy numbers in irrgs-CaM plants under both CO_2_ levels.

Changes in Ca^2+^ transients are an early event in plant response to diverse environmental signals. Previous studies found that higher Ca^2+^ concentration appears to be one of the most common physiological characteristics of plants in response to elevated CO_2_, which may be responsible for stomatal closure under elevated CO_2_ [36,37]. We found that elevated CO_2_ increased the calcium concentration in CMV-infected plant leaves and therefore upregulated the calmodulin protein rgs-CaM, which acted as part of a calcium signal transduction pathway. Furthermore, the rgs-CaM was able to recognize most RNA viruses, including the CMV, and degrade the VSR in the initial stage of infection [21]. Thus, the increase in Ca^2+^ concentration and downstream calmodulin would constitutively increase the resistance ability against CMV infection under elevated CO_2_. In summary, this study found that elevated CO_2_ increased Ca^2+^ concentration of CMV-infected plant and up-regulated gene expression of downstream calmodulin protein rgs-CaM, which would degrade the VSR protein 2b of CMV. The mRNA expression of 2b protein of CMV could be directly down-regulated under elevated CO_2_.Thus, elevated CO_2_ decreased the efficiency of CMV infection, which indicated that plants may suffer less CMV damage if atmospheric CO_2_ levels were to continue to increase. The outcomes of this study have important implications for agricultural virus control under anticipated future elevated CO_2_ conditions.

## Data Availability

No new data were created or analyzed in this study. Data sharing is not applicable to this article.

## Supplementary Materials

The following are available online at https://www.mdpi.com/2223-7747/10/1/59/s1, Figure S1: Structure of the pCAMBIA2301-RNAi binary destination vector for knocking-down rgs-CaM gene. This is a pCAMBIA2301 backbone-based vector with a GUS reporter sequence for further transgenic plant selection. Table S1: Primer sequences used for real-time quantitative PCR.

## References

[1] Solomon S., Qin D., Manning M., Chen Z., Marquis M., Averyt K.B., Tignor M., Miller H.L., IPCC (2007). Climate Change 2007: The physical science basis. Contribution of Working Group I to the Fourth Assessment Report of the Intergovernmental Panel on Climate Change.

[2] Ainsworth E.A., Long S.P. (2005). What have we learned from 15 years of free-air CO_2_ enrichment (FACE)? A meta-analytic review of the responses of photosynthesis, canopy properties and plant production to rising CO_2_. New Phytol..

[3] Ainsworth E.A., Leakey A.D., Ort D.R., Long S.P. (2008). FACE-ing the facts: Inconsistencies and interdependence among field, chamber and modeling studies of elevated [CO_2_] impacts on crop yield and food supply. New Phytol..

[4] Chakraborty S., Datta S. (2003). How will plant pathogens adapt to host plant resistance at elevated CO_2_ under a changing climate?. New Phytol..

[5] Zavala J.A., Nabity P.D., DeLucia E.H. (2013). An emerging understanding of mechanisms governing insect herbivory under elevated CO_2_. Annu. Rev. Entomol..

[6] Sun Y., Guo H., Ge F. (2016). Plant—Aphid interactions under elevated CO_2_: Some cues from aphid feeding behavior. Front. Plant Sci..

[7] Kazan K. (2018). Plant-biotic interactions under elevated CO_2_: A molecular perspective. Environ. Exp. Bot..

[8] Melloy P., Hollaway G., Luck J.O., Norton R.O.B., Aitken E., Chakraborty S. (2010). Production and fitness of Fusarium pseudograminearum inoculum at elevated carbon dioxide in FACE. Glob. Chang. Biol..

[9] Kobayashi T., Ishiguro K., Nakajima T., Kim H.Y., Okada M., Kobayashi K. (2006). Effects of elevated atmospheric CO_2_ concentration on the infection of rice blast and sheath blight. Phytopathology.

[10] Jwa N.S., Walling L.L. (2001). Influence of elevated CO_2_ concentration on disease development in tomato. New Phytol..

[11] Zhang S., Li X., Sun Z., Shao S., Hu L., Ye M., Shi K. (2015). Antagonism between phytohormone signalling underlies the variation in disease susceptibility of tomato plants under elevated CO_2_. J. Exp. Bot..

[12] Eastburn D.M., McElrone A.J., Bilgin D.D. (2011). Influence of atmospheric and climatic change on plant–pathogen interactions. Plant Pathol..

[13] Vaucheret H. (2006). Post-transcriptional small RNA pathways in plants: Mechanisms and regulations. Genes Dev..

[14] Ding S.W., Voinnet O. (2007). Antiviral immunity directed by small RNAs. Cell.

[15] Matros A., Amme S., Kettig B., Buck-sorlin G.H., Sonnewald U.W.E., Mock H.P. (2006). Growth at elevated CO_2_ concentrations leads to modified profiles of secondary metabolites in tobacco cv. SamsunNN and to increased resistance against infection with potato virus Y. Plant Cell Environ..

[16] Huang L., Ren Q., Sun Y., Ye L., Cao H., Ge F. (2012). Lower incidence and severity of tomato virus in elevated CO_2_ is accompanied by modulated plant induced defence in tomato. Plant Biol..

[17] Voinnet O. (2008). Use, tolerance and avoidance of amplified RNA silencing by plants. Trends Plant Sci..

[18] Vaucheret H. (2008). Plant argonautes. Trends Plant Sci..

[19] Bai M., Yang G.-S., Chen W.-T., Mao Z., Kang H.-X., Chen G.-H., Yang Y., Xie B. (2012). Genome-wide identification of Dicer-like, Argonaute and RNA-dependent RNA polymerase gene families and their expression analyses in response to viral infection and abiotic stresses in Solanum lycopersicum. Gene.

[20] Duan C.G., Fang Y.Y., Zhou B.J., Zhao J.H., Hou W.N., Zhu H., Ding S., Guo H.S. (2012). Suppression of Arabidopsis ARGONAUTE1-mediated slicing, transgene-induced RNA silencing, and DNA methylation by distinct domains of the Cucumber mosaic virus 2b protein. Plant Cell.

[21] Nakahara K.S., Masuta C., Yamada S., Shimura H., Kashihara Y., Wada T.S., Sekiguchi T. (2012). Tobacco calmodulin-like protein provides secondary defense by binding to and directing degradation of virus RNA silencing suppressors. Proc. Natl. Acad. Sci. USA.

[22] Gupta P., Duplessis S., White H., Karnosky D.F., Martin F., Podila G.K. (2005). Gene expression patterns of trembling aspen trees following long-term exposure to interacting elevated CO_2_ and tropospheric O_3_. New Phytol..

[23] Sun Y., Guo H., Zhu-Salzman K., Ge F. (2013). Elevated CO_2_ increases the abundance of the peach aphid on *Arabidopsis* by reducing jasmonic acid defenses. Plant Sci..

[24] Yu D., Liao L., Zhang Y., Xu K., Zhang J., Liu K., Li X., Tan G., Zheng J., He Y. (2018). Development of a Gateway-compatible pCAMBIA binary vector for RNAi-mediated gene knockdown in plants. Plasmid.

[25] Krügel T., Lim M., Gase K., Halitschke R., Baldwin I.T. (2002). Agrobacterium-mediated transformation of *Nicotiana attenuata*, a model ecological expression system. Chemoecology.

[26] Guo H., Gu L., Liu F., Chen F., Ge F., Sun Y. (2019). Phid-borne viral spread is enhanced by virus-induced accumulation of plant reactive oxygen species. Plant Physiol..

[27] Fang Y.Y., Zhao J.H., Liu S.W., Wang S., Duan C.G., Guo H.S. (2016). CMV2b-AGO interaction is required for the suppression of RDR-dependent antiviral silencing in Arabidopsis. Front. Microbiol..

[28] Schaad M.C., Jensen P., Carrington J.C. (1997). Formation of plant RNA virus replication complexes on membranes: Role of an endoplasmic reticulum-targeted viral protein. EMBO J..

[29] Brigneti G., Voinnet O., Wan-Xiang L., Ding S.W., Baulcombe D.C. (1998). Viral pathogenicity determinants are suppressors of transgene silencing. EMBO J..

[30] Zhang X.P., Liu D.S., Yan T., Fang X.D., Dong K., Xu J., Wang Y., Yu J., Wang X.B.L. (2017). Cucumber mosaic virus coat protein modulates the accumulation of 2b protein and antiviral silencing that causes symptom recovery in planta. PLoS Pathog..

[31] Candresse T., Hammond R.W., Hadidi A., Hadidi A., Khetarpal R.K., Koganezawa H. (1998). Detection and identification of plant viruses and viroids using polymerase chain reaction (PCR). Plant Virus Disease Control.

[32] Trębicki P., Nancarrow N., Cole E., Bosque-Pérez N.A., Constable F.E., Freeman A.J., Fitzgerald G.J. (2015). Virus disease in wheat predicted to increase with a changing climate. Glob. Chang. Biol..

[33] Liu J., Zhang X., Yang Y., Hong N., Wang G., Wang A., Wang L. (2016). Characterization of virus-derived small interfering RNAs in Apple stem grooving virus-infected in vitro-cultured *Pyrus pyrifolia* shoot tips in response to high temperature treatment. Virol. J..

[34] Paudel D.B., Sanfaçon H. (2018). Exploring the Diversity of Mechanisms Associated with Plant Tolerance to Virus Infection. Front. Plant Sci..

[35] Haxim Y., Ismayil A., Jia Q., Wang Y., Zheng X., Chen T., Cheng J. (2017). Autophagy functions as an antiviral mechanism against geminiviruses in plants. eLife.

[36] Assmann S.M. (1999). The cellular basis of guard cell sensing of rising CO_2_. Plant Cell Environ..

[37] Webb A.A., McAinsh M.R., Mansfield T.A., Hetherington A.M. (1996). Carbon dioxide induces increases in guard cell cytosolic free calcium. Plant J..

